# Checklist of the families Lonchopteridae and Phoridae of Finland (Insecta, Diptera)

**DOI:** 10.3897/zookeys.441.7197

**Published:** 2014-09-19

**Authors:** Jere Kahanpää

**Affiliations:** 1 Finnish Museum of Natural History, Zoology Unit, P.O. Box 17, FI-00014 University of Helsinki, Finland

**Keywords:** Checklist, Finland, Diptera, Lonchopteridae, Phoridae

## Abstract

A checklist of the Lonchopteridae and Phoridae recorded from Finland is presented.

## Introduction

The superfamily Phoroidea includes at least the fly families Phoridae, Lonchopteridae, and the small family Ironomyiidae known only from Australia. The flat-footed fly families Platypezidae and Opetiidae, treated in a separate paper in this volume, are placed either in their own basal superfamily Platypezoidea or included in Phoroidea (see Cumming et al. 1995, Woodley et al. 2009 and Wiegmann et al. 2011).

*Lonchoptera* Meigen, 1803 is the only currently recognized genus in Lonchopteridae. Five *Lonchoptera* species have been added to the Finnish fauna after the checklist of Hackman (1980) (Andersson 1991, Kahanpää 2013).

The scuttle flies (family Phoridae) may be the largest single family in Diptera. The true number of species in this family has been estimated to be over 30000, the vast majority still undescribed (Marshall 2012). The ‘megagenus’ *Megaselia* Rondani, 1856 contains about 50% of the species (Marshall 2012). Other phorid genera are relatively better known in Finland but none of them can be called well studied. It is likely that at least a hundred (or hundreds of) Finnish species remain unnoticed, most of them in *Megaselia*.

The checklist for *Megaselia* is mostly based on previously published records. The collections of Finnish Museum of Natural History (MZH) were examined by the author during the preparation of this checklist and a few obvious mistakes were taken into account. Most of the material in MZH was identified by H. Schmitz (between years 1915 and 1940) or E. Beyer (in 1958). The lists for other phorid genera are based on recently examined specimens from both MZH and private collections. Due to the difficulties encountered in identifying *Megaselia*, the list for this genus should definitely be considered preliminary. A revision of the Finnish material is sorely needed.

The Finnish species of these families were last listed by Hackman (1980).

**Number of species (Lonchopteridae, Phoridae):**

World: 63, 4105 species (Pape et al. 2011)

Europe: 13, 656 species

Finland: 8, ~224–234 species

Faunistic knowledge level in Finland: good, poor

## Checklist

suborder Brachycera Macquart, 1834

clade Eremoneura Lameere, 1906

clade Cyclorrhapha Brauer, 1863

superfamily Phoroidea Curtis, 1833

**LONCHOPTERIDAE** Macquart, 1835

***LONCHOPTERA*** Meigen, 1803

= ***Dipsa*** Fallén, 1810

*Lonchoptera bifurcata* (Fallén, 1810)

= *furcata* (Fallén, 1823)

*Lonchoptera fallax* de Meijere, 1906

*Lonchoptera impicta* Zetterstedt, 1848

*Lonchoptera lutea* Panzer, 1809

*Lonchoptera meijerei* Collin, 1938

*Lonchoptera nigrociliata* Duda, 1927

*Lonchoptera nitidifrons* Strobl, 1898

*Lonchoptera scutellata* Stein, 1890

**PHORIDAE** Curtis, 1833

***AENIGMATIAS*** Meinert, 1890

*Aenigmatias lubbockii* (Verrall, 1877)

***ANEVRINA*** Lioy, 1864

*Anevrina thoracica* (Meigen, 1804)

*Anevrina unispinosa* (Zetterstedt, 1860)

= *fennica* (Becker, 1901)

*Anevrina urbana* (Meigen, 1830)

***BOROPHAGA*** Enderlein, 1924

*Borophaga agilis* (Meigen, 1830)

*Borophaga carinifrons* (Zetterstedt, 1848)

*Borophaga femorata* (Meigen, 1830)

*Borophaga incrassata* (Meigen, 1830)

*Borophaga subsultans* (Linnaeus, 1767)

= *okellyi* Schmitz, 1937

***CHAETOPLEUROPHORA*** Schmitz, 1922

*Chaetopleurophora erythronota* (Strobl, 1892)

*Chaetopleurophora spinosissima* (Strobl, 1892)

***CONICERA*** Meigen, 1830

*Conicera dauci* (Meigen, 1830)

= *atra* Meigen, 1830

*Conicera floricola* Schmitz, 1938

= *similis* auct. nec (Haliday, 1833)

= *minuscula* Schmitz, 1953

*Conicera schnittmanni* Schmitz, 1926

*Conicera similis* (Haliday, 1833)

= *pauxilla* Schmitz, 1920

? *Conicera tarsalis* Schmitz, 1920

*Conicera tibialis* Schmitz, 1925

= *fallens* Schmitz, 1948

***DIPLONEVRA*** Lioy, 1864

= ***Diploneura*** emend.

*Diplonevra florescens* (Turton, 1801)

= *florea* (Fabricius, 1794) preocc.

= *abdominalis* (Fallén, 1823)

*Diplonevra concinna* (Meigen, 1830)

*Diplonevra freyi* Schmitz, 1927

*Diplonevra funebris* (Meigen, 1830)

= *rostralis* (Schmitz, 1918)

*Diplonevra glabra* Schmitz, 1927

= *parcepilosa* Schmitz, 1927

*Diplonevra nitidula* (Meigen, 1830)

*Diplonevra oldenbergi* Schmitz, 1920

***DOHRNIPHORA*** Dahl, 1898

*Dohrniphora cornuta* (Bigot, 1857)

***GYMNOPHORA*** Macquart, 1835

*Gymnophora arcuata* (Meigen, 1830)

*Gymnophora healeyae* Disney, 1980

*Gymnophora nigripennis* Schmitz, 1926

*Gymnophora quartomollis* Schmitz, 1920

***HYPOCERA*** Lioy, 1864

*Hypocera mordellaria* (Fallén, 1823)

***MEGASELIA*** Rondani, 1856

= ***Aphiochaeta*** Brues, 1903

= ***Plastophora*** Brues, 1905

*Megaselia aequalis* (Wood, 1909)

*Megaselia affinis* (Wood, 1909)

= *proxima* (Lundbeck, 1920)

*Megaselia albicaudata* (Wood, 1910)

*Megaselia albiclava* (Schmitz, 1926)

*Megaselia altifrons* (Wood, 1909)

*Megaselia analis* (Lundbeck, 1920)

? *Megaselia angelicae* (Wood, 1910)

*Megaselia angularis* (Schmitz, 1924)

*Megaselia angusta* (Wood, 1909)

= *angustata* emend.

= *pulicaria* auct. nec (Fallén, 1923)

*Megaselia annulipes* (Schmitz, 1921)

*Megaselia armata* (Wood, 1909)

*Megaselia atrosericea* Schmitz, 1927

*Megaselia auricoma* Schmitz, 1927

*Megaselia baltica* (Schmitz, 1924)

*Megaselia barbulata* (Wood, 1909)

= *depilata* (Lundbeck, 1921)

? *Megaselia basispinata* (Lundbeck, 1920)

*Megaselia basiveluta* Schmitz, 1935

*Megaselia beckeri* (Wood, 1909)

= *laticrus* Schmitz, 1927

*Megaselia berndseni* (Schmitz, 1919)

= *pygmaeoides* (Lundbeck, 1921)

*Megaselia bovista* (Gimmerthal, 1848)

*Megaselia brevicostalis* (Wood, 1910)

*Megaselia breviseta* (Wood, 1912)

*Megaselia breviterga* (Lundbeck, 1920)

= *similata* (Lundbeck, 1921)

*Megaselia campestris* (Wood, 1908)

*Megaselia ciliata* (Zetterstedt, 1848)

*Megaselia cinereifrons* (Strobl, 1910)

*Megaselia clara* (Schmitz, 1921)

*Megaselia coaetanea* Schmitz, 1929

*Megaselia coccyx* Schmitz, 1965

? *Megaselia collini* (Wood, 1909)

*Megaselia conformis* (Wood, 1909)

*Megaselia costalis* (von Roser, 1840)

*Megaselia curvivenia* Schmitz, 1928

*Megaselia dahli* (Becker, 1901)

*Megaselia discreta* (Wood, 1909)

= *nudiventris* (Wood, 1909)

*Megaselia diversa* (Wood, 1909)

*Megaselia dubitalis* (Wood, 1908)

*Megaselia eccoptomera* Schmitz, 1927

*Megaselia eisfelderae* Schmitz, 1948

*Megaselia elongata* (Wood, 1914)

*Megaselia erecta* (Wood, 1910)

*Megaselia exarcuata* Schmitz, 1927

*Megaselia excavata* Schmitz, 1927

*Megaselia excorticata* Disney, 2009

*Megaselia fennicola* Beyer, 1958

*Megaselia flammula* Schmitz, 1928

*Megaselia flava* (Fallén, 1823)

*Megaselia flavicoxa* (Zetterstedt, 1848)

*Megaselia fumata* (Malloch, 1909)

*Megaselia funeralis* Schmitz, 1928

*Megaselia fungivora* (Wood, 1909)

*Megaselia fusca* (Wood, 1909)

*Megaselia fuscipalpis* (Lundbeck, 1920)

*Megaselia fuscoides* Schmitz, 1934

*Megaselia fuscovariana* Schmitz, 1933

*Megaselia giraudii* (Egger, 1862)

*Megaselia glabrifrons* (Wood, 1909)

*Megaselia groenlandica* (Lundbeck, 1901)

*Megaselia halterata* (Wood, 1910)

= *plurispinosa* (Lundbeck, 1920)

*Megaselia hectochaeta* Schmitz, 1974

*Megaselia hilaris* Schmitz, 1927

*Megaselia hirsuta* (Wood, 1910)

*Megaselia hirticaudata* (Wood, 1910)

*Megaselia hirticrus* (Schmitz, 1918)

*Megaselia humeralis* (Zetterstedt, 1838)

= *cubitalis* (Becker, 1901)

*Megaselia hyalipennis* (Wood, 1912)

*Megaselia infraposita* (Wood, 1909)

*Megaselia ignobilis* (Schmitz, 1919)

*Megaselia intonsa* Schmitz, 1948

*Megaselia involuta* (Wood, 1910)

*Megaselia latifemorata* (Becker, 1901)

*Megaselia limburgensis* (Schmitz, 1918)

*Megaselia longicostalis* (Wood, 1912)

*Megaselia longifurca* (Lundbeck, 1921)

= *spinolabella* Disney, 1989

*Megaselia longipalpis* (Wood, 1910)

*Megaselia longiseta* (Wood, 1909)

*Megaselia lucifrons* (Schmitz, 1918)

= *subnitida* (Lundbeck, 1920)

*Megaselia luminifrons* (Schmitz, 1926)

*Megaselia lutea* (Meigen, 1830)

*Megaselia major* (Wood, 1912)

*Megaselia mallochi* (Wood, 1909)

*Megaselia manicata* (Wood, 1910)

*Megaselia maura* (Wood, 1910)

*Megaselia meconicera* (Speiser, 1925)

*Megaselia meigeni* (Becker, 1901)

? *Megaselia miki* Schmitz, 1929

*Megaselia minor* (Zetterstedt, 1848)

= *angustifrons* (Wood, 1912)

*Megaselia minuta* (Aldrich, 1892)

= *luminosa* Schmitz, 1952

*Megaselia mixta* (Schmitz, 1918)

*Megaselia nasoni* (Malloch, 1914)

= *coaequalis* (Schmitz, 1919)

*Megaselia nigra* (Meigen, 1830)

= *albidihalteralis* (Felt, 1896)

*Megaselia nigriceps* (Loew, 1866)

= *projecta* (Becker, 1901)

*Megaselia nigripalpis* (Lundbeck, 1920)

*Megaselia nudipleura* (Beyer, 1958)

*Megaselia obscuripennis* (Wood, 1909)

*Megaselia offuscata* (Schmitz, 1921)

*Megaselia opacicornis* Schmitz, 1949

*Megaselia parnassia* Disney, 1986

*Megaselia parva* (Wood, 1909)

*Megaselia pectoralis* (Wood, 1910)

*Megaselia pectunculata* Schmitz, 1927

*Megaselia picta* (Lehmann, 1822)

*Megaselia pleuralis* (Wood, 1909)

*Megaselia plurispinulosa* (Zetterstedt, 1860)

*Megaselia posticata* (Strobl, 1898)

*Megaselia praeacuta* (Schmitz, 1919)

*Megaselia prodroma* (Lundbeck, 1921)

*Megaselia producta* (Schmitz, 1921)

= *sordescens* auct. nec Schmitz, 1927

*Megaselia propinqua* (Wood, 1909)

*Megaselia pubecula* Schmitz, 1927

*Megaselia pulicaria* (Fallén, 1923)

= *sinuata* Schmitz, 1926

*Megaselia pumila* (Meigen, 1830)

= *atripes* (Brues, 1915)

*Megaselia pusilla* (Meigen, 1830)

*Megaselia pygmaea* (Zetterstedt, 1848)

*Megaselia quadriseta* (Schmitz, 1918)

= *badia* Schmitz, 1938

? *Megaselia raetica* Schmitz, 1934

*Megaselia robertsoni* Disney, 2008

*Megaselia robusta* Schmitz, 1928

*Megaselia rubricornis* (Schmitz, 1919)

*Megaselia rudis* (Wood, 1909)

*Megaselia ruficornis* (Meigen, 1830)

*Megaselia rufipes* (Meigen, 1804)

*Megaselia scalaris* (Loew, 1866)

*Megaselia scutellaris* (Wood, 1909)

= *scutellariformis* (Schmitz, 1926)

*Megaselia sepulchralis* (Lundbeck, 1920)

*Megaselia setulipalpis* Schmitz, 1938

*Megaselia sordida* (Zetterstedt, 1838)

= *eminens* Schmitz, 1953

= *semiscaura* Schmitz, 1927

? *Megaselia specularis* Schmitz, 1935

*Megaselia spinicincta* (Wood, 1910)

*Megaselia spinigera* (Wood, 1908)

*Megaselia styloprocta* (Schmitz, 1921)

*Megaselia subcarpalis* (Lundbeck, 1920)

*Megaselia subpalpalis* (Lundbeck, 1920)

*Megaselia subpleuralis* (Wood, 1909)

*Megaselia subtumida* (Wood, 1909)

*Megaselia sulphuripes* (Meigen, 1830)

*Megaselia superciliata* (Wood, 1910)

*Megaselia superfurcata* Schmitz, 1928

*Megaselia sylvatica* (Wood, 1910)

= *impolluta* (Schmitz, 1920)

*Megaselia tarsalis* (Wood, 1910)

*Megaselia tenuiventris* Schmitz, 1927

*Megaselia tignorum* Disney, 2009

*Megaselia tumida* (Wood, 1909)

= *setifer* Lundbeck, 1920

*Megaselia unicolor* (Schmitz, 1919)

*Megaselia valvata* Schmitz, 1935

*Megaselia variana* Schmitz, 1926

*Megaselia vestita* (Wood, 1914)

*Megaselia winqvisti* Disney, 2011

*Megaselia woodi* (Lundbeck, 1922)

*Megaselia xanthozona* (Strobl, 1892)

= *euryprocta* Schmitz, 1957

*Megaselia zonata* (Zetterstedt, 1838)

***MENOZZIOLA*** Schmitz, 1927

*Menozziola obscuripes* (Schmitz, 1927)

***METOPINA*** Macquart, 1835

*Metopina galeata* (Haliday, 1833)

= *inaequalis* Schmitz, 1927

*Metopina oligoneura* (Mik, 1867)

*Metopina pileata* Schmitz, 1936

***MICROSELIA*** Schmitz, 1934

*Microselia forsiusi* (Schmitz, 1927)

***PHALACROTOPHORA*** Enderlein, 1912

*Phalacrotophora berolinensis* Schmitz, 1920

*Phalacrotophora beuki* Disney in Disney & Beuk, 1997

*Phalacrotophora fasciata* (Fallén, 1823)

***PHORA*** Latreille, 1796

= ***Trineura*** Meigen, 1803

*Phora artifrons* Schmitz, 1920

*Phora atra* (Meigen, 1804)

= *aterrima* (Fabricius, 1794) preocc.

*Phora bullata* Schmitz, 1927

*Phora convallium* Schmitz, 1928

*Phora convergens* Schmitz, 1920

*Phora dubia* (Zetterstedt, 1848)

= *schineri* (Becker, 1901)

*Phora edentata* Schmitz, 1920

*Phora hamata* Schmitz, 1927

*Phora holosericea* Schmitz, 1920

*Phora hyperborea* Schmitz, 1927

*Phora obscura* (Zetterstedt, 1848)

*Phora occidentata* Malloch, 1912

= *zetterstedti* Schmitz, 1927

*Phora penicillata* Schmitz, 1920

*Phora praepandens* Schmitz, 1927

*Phora pubipes* Schmitz, 1920

*Phora stictica* Meigen, 1830

*Phora tincta* Schmitz, 1920

***PLECTANOCNEMA*** Schmitz, 1926

*Plectanocnema nudipes* (Becker, 1901)

***PSEUDACTEON*** Coquillett, 1907

*Pseudacteon fennicus* Schmitz, 1927

*Pseudacteon formicarum* (Verrall, 1877)

***SPINIPHORA*** Malloch, 1909

*Spiniphora bergenstammi* Mik, 1864

*Spiniphora dorsalis* (Becker, 1901)

*Spiniphora excisa* (Becker, 1901)

*Spiniphora maculata* (Meigen, 1830)

= *helicivora* (Dufour, 1841)

***TRIPHLEBA*** Rondani, 1856

= ***Citrago*** Schmitz, 1924

*Triphleba admirabilis* Schmitz, 1927

? *Triphleba aequalis* (Schmitz, 1919)

? *Triphleba autumnalis* (Becker, 1901)

*Triphleba bicornuta* (Strobl, 1910)

= *uncinata* (Schmitz, 1918)

*Triphleba citreiformis* (Becker, 1901)

*Triphleba distinguenda* (Strobl, 1892)

= *unicalcarata* (Becker, 1901)

*Triphleba excisa* (Lundbeck, 1921)

*Triphleba gracilis* (Wood, 1907)

*Triphleba hyalinata* (Meigen, 1830)

? *Triphleba inaequalis* Schmitz, 1943

*Triphleba intermedia* (Malloch, 1908)

*Triphleba lugubris* (Meigen, 1830)

*Triphleba luteifemorata* (Wood, 1906)

*Triphleba nudipalpis* (Becker, 1901)

*Triphleba opaca* (Meigen, 1830)

*Triphleba pachyneurella* (Schmitz, 1919)

*Triphleba palposa* (Zetterstedt, 1848)

*Triphleba renidens* Schmitz, 1927

*Triphleba subcompleta* Schmitz, 1927

*Triphleba trinervis* (Becker, 1901)

***VERUANUS*** Schmitz, 1927

*Veruanus oldenbergi* Schmitz, 1919

= *memorabilis* Schmitz, 1927

## Excluded species

*Megaselia cirriventris* Schmitz, 1929 mistake?

*Megaselia crassipes* (Wood, 1909) misidentified

*Megaselia digitalis* Schmitz, 1957 mistake?

*Megaselia indifferens* (Lundbeck, 1920) not found within present borders

*Megaselia lapponica* Schmitz, 1928 not found within present borders

*Megaselia luteipes* (Schmitz, 1918) misidentified

*Megaselia palmeni* (Becker, 1901) not found within present borders

*Megaselia septentrionalis* (Schmitz, 1919) not found within present borders

*Megaselia simulans* (Wood, 1912) not found within present borders ?

*Triphleba gilvipes* Schmitz, 1943 not found within present borders

## Notes

***Conicera tarsalis***, ***Megaselia collini***. Records of these species are based on single females identified by H. Schmitz.

***Megaselia basispinata***, ***Megaselia miki***, ***Megaselia raetica***. Species recorded from Finland by Hackman (1980) and Disney (1991) but no Finnish specimens or published records with details could be located.

***Megaselia cirriventris***, ***Megaselia digitalis***. These species were recorded from ‘Fennia’ and ‘Lappland’ by Schmitz (1958), but all specimens listed are from the Abisko region in northen Sweden. It seems likely that Schmitz erroneously assigned the material from Swedish Lappland to Finland.

***Megaselia luteipes***. Examined specimens belong to Megaselia sp. not luteipes (females) (Espoo, leg. W. Hackman) and possibly *Megaselia* sp. 24 of Disney (2013) (Finland, Muonio, leg. J. Sahlberg and Russia, Kola, Kantalahti, leg. R. Frey).

***Megaselia pulicaria* agg**. This difficult complex of species was revised by Disney (1999). The Finnish material has not been re-examined. The Finnish species involved are *Megaselia angusta*, *Megaselia costalis*, *Megaselia longicostalis*, *Megaselia longifurca*, *Megaselia pulicaria*, *Megaselia subtumida* and *Megaselia tumida*.

***Megaselia simulans***. Recorded only(?) from Kuusamo, a municipality split between Finland and Russia in 1944. Most records preceding the split are from the Russian side. No material was found in MZH.

***Megaselia specularis***. Recorded, with some doubt, from Finland by Hackman (1963). No material could be found in MZH.

***Triphleba aequalis***. Schmitz (1943) mention a Finnish male stored in MZH, but no such specimen could be found.

***Triphleba autumnalis***, ***Triphleba inaequalis***. Finnish according to Disney (1991), but no specimens or detailed records could be located.
